# Clinical characteristics of hospitalized children with mycoplasma pneumoniae pneumonia in Chinese tertiary hospitals during the 2023–2024 post-pandemic period

**DOI:** 10.3389/fcimb.2026.1652640

**Published:** 2026-03-24

**Authors:** Xiancheng Wang, Xiaoshun Zheng, Longfei Wang, Lijuan Zhang, Yuming Xu, Shuang Deng, Lihao Li, Jie He

**Affiliations:** 1 Department of Pediatrics, Shenzhen Hospital of Southern Medical University, Shenzhen, China; 2 Department of Neurosurgery, The Sixth Affiliated Hospital, Sun Yat-sen University, Guangzhou, China

**Keywords:** epidemiology, laboratory-clinical discordance, *M. pneumoniae*, macrolide-resistance, pneumonia, post-pandemic

## Abstract

**Backgrounds:**

The COVID-19 pandemic and subsequent non-pharmaceutical interventions (NPIs) altered the epidemiological patterns of *Mycoplasma pneumoniae (M. pneumoniae)* infections. This study aimed to analyze the long-term trends of *M. pneumoniae* pneumonia (MPP) in children after NPI discontinuation, focusing on epidemiological, clinical and macrolide-resistant characteristics.

**Methods:**

A retrospective analysis was conducted on 1342 hospitalized children with MPP from January 2023 to December 2024 in Shenzhen Hospital of Southern Medical University, China. MPP, macrolide-unresponsive MPP (MUMPP), and severe MPP (SMPP) were diagnosed according to predefined criteria. Differences between mutated MPP and MUMPP were investigated, and risk factors for MUMPP, SMPP and mixed infection were assessed.

**Results:**

MPP exhibits a bimodal temporal distribution in 2023-2024, with the first peak occurring in November 2023, and the second smaller peak in August 2024. The macrolide resistance gene positive rate was 86.43% (121/140), but only 64.29% (90/140) were clinically unresponsive to macrolides (*P* < 0.0001). Risk factors for MUMPP included older age, longer hospital stay, and severe cough. Approximately 25% of MPP cases progressed to SMPP, with younger age, prolonged cough, and higher inflammatory markers being associated with severity. Co-pathogen infections were common, and patients with mixed infections had distinct clinical and laboratory profiles compared to those with pure MPP.

**Conclusions:**

This study highlights the complex epidemiological and clinical landscape of MPP in the post-pandemic era. The discordance between macrolide resistance genes and clinical treatment suggests that macrolides may still be effective in some cases. The identified risk factors and clinical features provide valuable insights for optimizing treatment strategies and managing pediatric MPP in the post-pandemic context.

## Introduction

Community-acquired pneumonia (CAP) remains a critical global public health concern in pediatric populations (Walker et al., 2013; Marangu and Zar, 2019; Meyer Sauteur, 2024). In China, *Mycoplasma pneumoniae* has emerged as one of the most predominant pathogens of CAP, with a detection rate of 19.16% among children (Zhu et al., 2018). Notably, this pathogen is particularly prevalent in school-aged children and adolescents (Ding et al., 2024).

The implementation of non-pharmaceutical interventions (NPIs) during the COVID-19 pandemic since March 2020 has profoundly altered the epidemiological patterns of respiratory pathogens, including *M. pneumoniae* (Meyer Sauteur and Beeton, 2024a). Following the gradual relaxation of NPIs, a remarkable resurgence of *M. pneumoniae* infections has been observed globally. The ESGMAC MAPS study, as the first global prospective surveillance study of *M. pneumoniae* initiated in April 2022, documented a significant outbreak of *M. pneumoniae* infection in 2023 (Meyer Sauteur and Beeton, 2024a). This resurgence has been characterized by several novel clinical and epidemiological features (Xie et al., 2024; Yan et al., 2024), including increased macrolide resistance rates, higher incidence of severe cases, more frequent coinfections, greater proportion of infections in children under 3 years, and elevated occurrence of hypoxemia. However, current studies have mainly analyzed short-term data (spanning months to one year), leaving a gap in understanding the long-term trends after the COVID-19 pandemic.

Our study would comprehensively analyze clinical data from two full years (2023-2024) following NPI discontinuation, aiming to address: (1) the epidemiological and clinical characteristics of *M. pneumoniae* infections; (2) the evolving macrolide-resistant patterns of *M. pneumoniae* pneumonia (MPP), and (3) the implications for optimizing clinical treatment strategies. Our findings will provide evidence-based insights for the management and precision treatment of pediatric *M. pneumoniae* infections in the post-pandemic era.

## Methods

### Participants and definitions of clinical outcomes

We conducted a retrospective analysis of pediatric patients hospitalized with MPP at Shenzhen Hospital of Southern Medical University between January 1, 2023, and December 31, 2024.

According to the Chinese Guidelines for the diagnosis and treatment of MPP in children (2023 edition) (National Health Commission of the People’s Republic of China. Guidelines for the diagnosis and treatment of Mycoplasma pneumoniae pneumonia in children (2023 edition), 2023), pediatric MPP were diagnosed if they met all the following criteria: (i) fever, or acute respiratory symptoms (cough, tachypnea, difficulty breathing) or both; (ii) low breathing or dry, wet rales; (iii) chest film findings characterized by lung portal lymph node and lung gate shadow, bronchopneumonia, interstitial pneumonia, and large and high-density shadow; (iv) confirmed by positive polymerase chain reaction (PCR) result of *M. pneumoniae*-DNA. Specifically, diagnostic confirmation was strictly limited to the detection of *M. pneumoniae* DNA, excluding serological evidence (IgM/IgG antibodies) or antigen detection. Patients were excluded if they had comorbidities potentially confounding pneumonia severity (bronchopulmonary dysplasia, congenital heart disease, immunodeficiency); suspected hospital-acquired or ventilator-associated pneumonia; incomplete clinical records.

According to the Chinese Guidelines for the diagnosis and treatment of MPP in children (2023 edition) (National Health Commission of the People’s Republic of China. Guidelines for the diagnosis and treatment of Mycoplasma pneumoniae pneumonia in children (2023 edition), 2023), macrolide-unresponsive MPP (MUMPP) is defined as MPP in children who, after 72 hours of standard treatment with macrolide antibiotics (including azithromycin, cephalosporin, penicillin, etc.), continue to have persistent fever, with no improvement or further aggravation of clinical signs and pulmonary imaging findings.

Severe MPP (SMPP) refers to MPP with severe condition meeting any of the following criteria.: (i) sustained high fever (≥39 °C) for ≥5 days or fever for ≥7 days without a downward trend in peak body temperature; (ii) presence of at least one of the following: wheezing, shortness of breath, dyspnea, chest pain, or hemoptysis; (iii) development of extrapulmonary complications; (iv) resting oxygen saturation (SpO_2_) ≤93%; (v) imaging findings demonstrated by either: (a) involvement of ≥2/3 of a single lung lobe with homogeneous high-density consolidation, or high-density consolidation in ≥2 lung lobes (regardless of involvement area), with or without moderate-to-large pleural effusion or focal bronchiolitis; (b) diffuse involvement of a single lung or bronchiolitis in ≥4/5 of bilateral lung lobes, with possible coexisting bronchitis and mucus plugging leading to atelectasis; (vi) progression of clinical symptoms and imaging evidence of lesion expansion by >50% within 24–48 hours; (vii) significant elevation of C-reactive protein (CRP), lactate dehydrogenase (LDH), or D-dimer, according to the Chinese Guidelines for the diagnosis and treatment of MPP in children (2023 edition) (National Health Commission of the People’s Republic of China. Guidelines for the diagnosis and treatment of Mycoplasma pneumoniae pneumonia in children (2023 edition), 2023).

This study was approved by the Ethics Committee of Shenzhen Hospital of Southern Medical University (Approval No: NYSZYYEC2024K064R001). Since this was a retrospective study and no human or animal experiments were conducted, informed consents were waived by the Ethics Committee of Shenzhen Hospital of Southern Medical University.

### Detection of *M. pneumoniae* and other co-pathogens

Nasopharyngeal swabs were collected from all patients within 24 hours after admission in strict accordance with established clinical procedures by trained doctors. PCR-flow fluorescence method was employed for screening 18 common respiratory pathogens by a commercially available kit (manufactured by Huayin Health Co., Ltd., Guangzhou, China), following the recommended guidelines. The 18 common respiratory pathogens included *M. pneumoniae* (MP), adenovirus (AdV), cytomegalovirus (CMV), Epstein-Barr virus (EBV), enterovirus (EV), human bocavirus (hBoV), human herpesvirus 6 (HHV6), human metapneumovirus (hMPV), human rhinovirus (HRV), infuenza virus A (IFA), infuenza virus B (IFB), parainfuenza virus (PIV), respiratory syncytial virus (RSV), Severe Acute Respiratory Syndrome Coronavirus 2 (SARS-CoV-2), *Bordetella pertussis* (BP), *Haemophilus influenzae* (HI), *Moraxella catarrhalis* (MC), *Streptococcus pneumoniae* (SP). Nucleic acid extraction was performed using a fully automated high-throughput nucleic acid extractor (MagMix 96; Guangzhou Magen Biotechnology Co., Ltd., Guangzhou, China). Subsequently, nucleic acid amplification was carried out with an amplification instrument (TC-96/G/H(b); Hangzhou Bioer Technology Co., Ltd., Hangzhou, China). Detection of respiratory pathogens was accomplished by a liquid-phase suspension chip detector (MAGPIX; Luminex Corporation, Austin, TX, USA).

### Targeted next-generation sequencing detection

Among the 1342 cases of *M. pneumoniae* infection, a total of 140 throat swab samples were collected for tNGS detection, aiming to identify respiratory pathogens and detect macrolide resistance mutations. Patients who opted for tNGS testing tended to have greater clinical severity, prolonged illness, or higher socioeconomic status. The tNGS service was provided by a commercial company laboratory (KingMed Diagnostics, Guangzhou, China). According to the manufacturer’s specifications, this method enables the early diagnosis of respiratory infection by simultaneously detecting 198 pathogens including 80 bacteria, 79 viruses (35 DNA viruses and 44 RNA viruses), 32 fungi, *M. pneumoniae*, *Chlamydia pneumoniae*, *Chlamydia trachomatis*, *Chlamydia psittaci*, *Ureaplasma parvos*, *Ureaplasma urealyticum*, and *Benakodia*. Mutations at the 23S rRNA region of *M. pneumoniae* at A2063G, A2064G, A2067G, and C2617G were also examined. Patients found to carry macrolide resistance mutations in the 23S rRNA gene were considered as mutated MPP.

### Data collections

Clinical information, including patients’ sex, age, symptoms and signs (days of fever, fever (>39°C), hyperpyrexia (>40°C), days of cough, bad cough, wheezing, three concave sign, asymmetric lung sounds, rales, pleural effusion, pulmonary consolidation), extrapulmonary complications (gastrointestinal dysfunction, rash), treatment (bronchoscopy therapy, oxygen therapy, antibacterial therapy, methylprednisolone) and length of hospital stay were collected retrospectively from patient records.

Laboratory tests including white blood cell (WBC) counts, neutrophil (NEUT) counts, lymphocyte (LYMPH) counts, erythrocyte (EO) counts, red blood cell (RBC) counts, hemoglobin (HGB), red blood cell distribution width - coefficient of variation (RDW-CV), red blood cell distribution width - standard deviation (RDW-SD), platelet (PLT) counts, high-sensitivity C-reactive protein (hs-CRP), procalcitonin (PCT), interleukin-6 (IL-6), lactate dehydrogenase (LDH), aspartate transaminase (AST), alanine transaminase (ALT), erythrocyte sedimentation rate (ESR) were measured within 24 h after admission.

### Statistical analysis

Continuous characteristics were analyzed using t-tests and reported as means ± SD. Categorical variables were compared by the chi-square test and presented as numbers and percentage. Multivariable logistic regressions were used to assess the associations of various clinical and laboratory variables with MUMPP, SMPP and mixed infection. After exclusion of variables with severe collinearity (VIF>10), variables with *P* < 0.20 in Chi-square tests or t-tests were included in the multivariable models, while child’s sex and age were forced in the model even if above the selection limit, because both of the factors were strongly associated with clinical outcomes and therefore commonly adjusted as covariates in previous studies. Matched Chi-square test was applied to investigated the difference between mutated MPP and MUMPP. A two-tailed *P* value < 0.05 was considered statistically significant. All statistical analyses were performed using R software (version 4.4.2).

## Results

### Study population and temporal distribution of MPP

**Figure 1:**
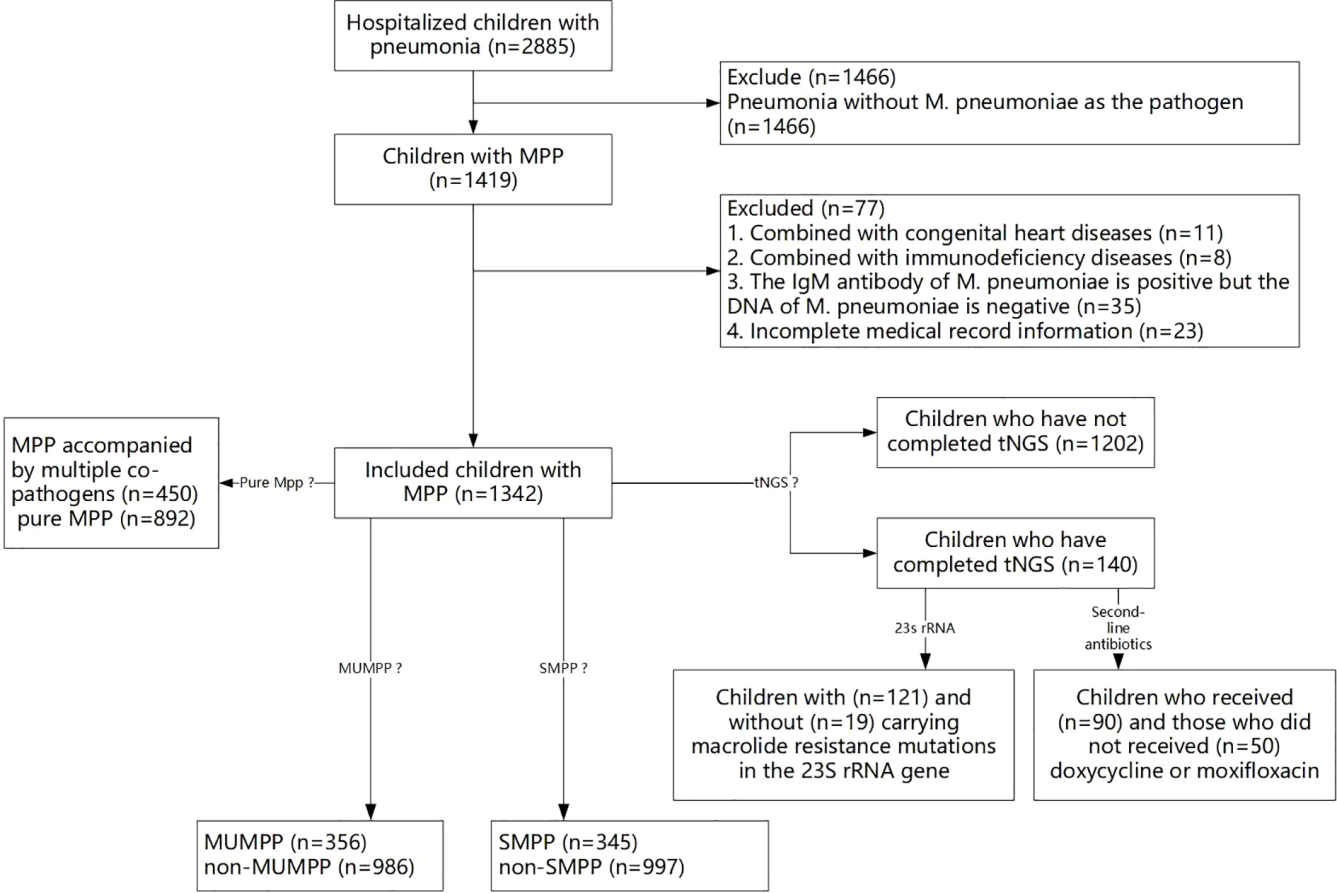
The flow chart of the study population.

From January 2023 to December 2024, 2885 hospitalized children with pneumonia were enrolled, among whom 1419 (49.19%, 1419/2885) children were diagnosed as MPP. After excluded children: (1) combined with congenital heart diseases (n=11); (2) combined with immunodeficiency diseases (n=8); (3) the IgM antibody of *M. pneumoniae* is positive but the DNA of *M. pneumoniae* is negative (n=35); (4) with incomplete medical record information (n=23), a total of 1342 children with MPP were included in the present study. A flow chart of the study population is shown in **Figure 1**.

**Figure 2:**
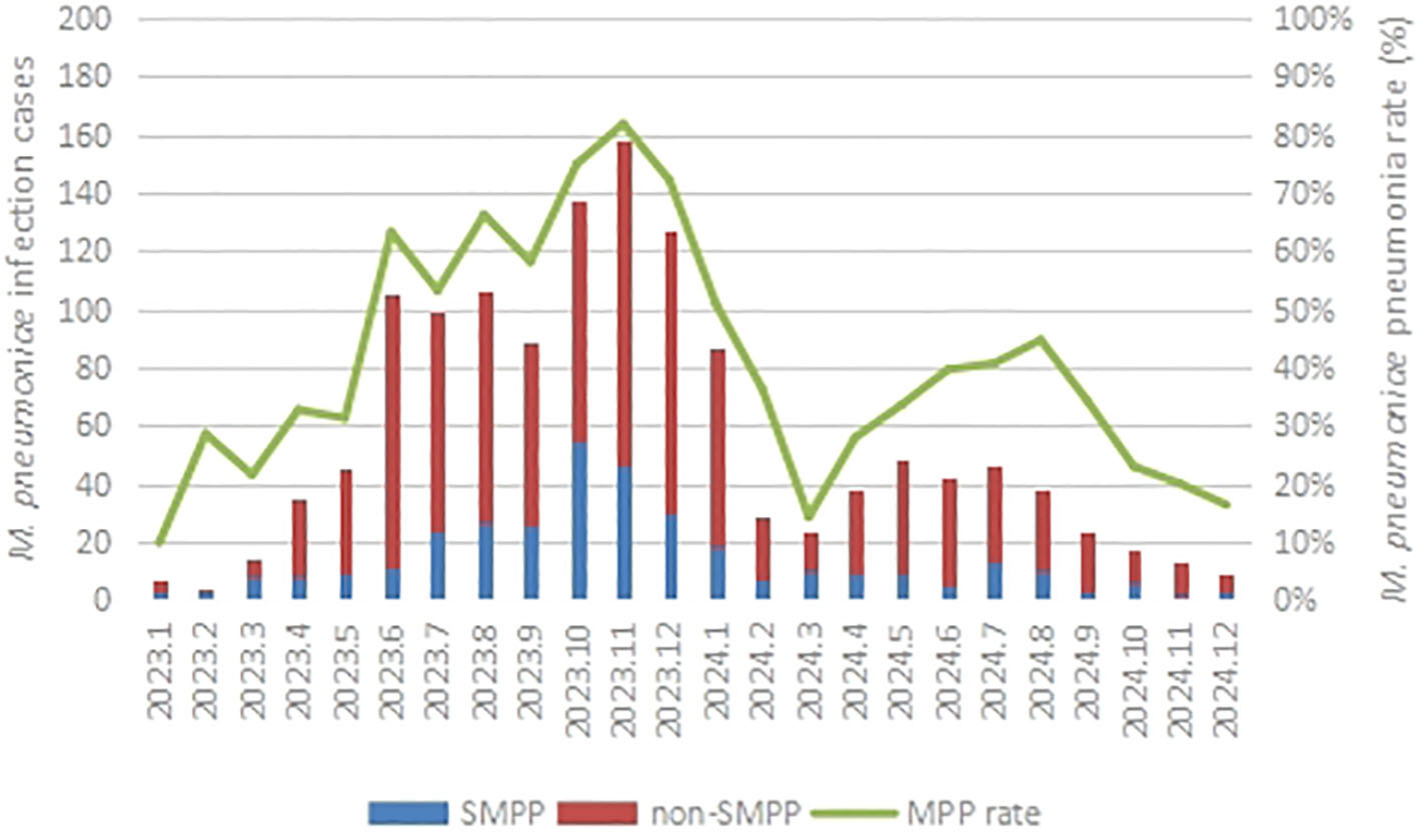
The monthly distributions of M. pneumoniae pneumonia from January 2023 to December 2024.

As shown in **Figure 2**, the first peak of the MPP positive rate was observed from June 2023 to January 2024, with the rate varying from 50.88% (January 2024, 87/171) to 81.87% (November 2023, 158/193), followed by a relatively lower peak emerging from May 2024 to September 2024, with the positive rate ranging from 33.57% (May 2024, 48/143) to 44.71% (August 2024, 38/85).

### Comparison of patients with MUMPP and non-MUMPP

Among the 1342 cases of *M. pneumoniae* infection, 986 patients were administered azithromycin and/or cephalosporin and/or penicillin, whereas the other 356 were treated with doxycycline or moxifloxacin. The former was considered to be non-MUMPP, while the latter was considered to have MUMPP.

The clinical characteristics and laboratory findings of hospitalized patients with MUMPP and non-MUMPP were compared (**Table 1**). Patients with clinical MUMPP were older, more likely to present with fever, hyperpyrexia, bad cough, asymmetric lung sounds, pleural effusion, pulmonary consolidation, gastrointestinal dysfunction, and a longer length of hospital stay (all *P* < 0.05). Moreover, patients with MUMPP were more likely to have lower levels LYMPH, PLT, ESR, and higher hs-CRP, and were more likely to be treated with oxygen therapy, and methylprednisolone (all *P* < 0.05). No significant difference in sex was observed between the MUMPP and non-MUMPP groups (*P*>0.05). In addition, no significant differences in the rates of severe pneumonia and mixed infection were observed between the MUMPP and non-MUMPP groups (*P*>0.05).

**Table 1:** Clinical and laboratory characteristics of patients with MUMPP and non-MUMPP.

	Total (N = 1342)	MUMPP (N = 356)	non-MUMPP (N = 986)	*P* value
Sex (male)	698 (52.01)	174 (48.88)	524 (53.14)	0.167
Age (months)	6.2 ± 2.9	7.1 ± 2.8	5.9 ± 2.9	**<0.001**
<=1	41 (3.06)	4 (1.12)	37 (3.75)	
1~3	287 (21.39)	47 (13.2)	240 (24.34)	
4~6	471 (35.1)	118 (33.15)	353 (35.8)	
>=7	543 (40.46)	187 (52.53)	356 (36.11)	
Symptoms and signs				
Days of fever	4.9 ± 2.6	5.1 ± 2.5	4.9 ± 2.6	0.131
Fever (>39°C)	918 (68.41)	265 (74.44)	653 (66.23)	**0.004**
Hyperpyrexia (>40°C)	288 (21.54)	90 (25.28)	198 (20.18)	**0.045**
Days of cough	7.3 ± 7.3	7.7 ± 8.3	7.2 ± 6.9	0.249
Bad cough	499 (37.18)	166 (46.63)	333 (33.77)	**<0.001**
Wheezing	122 (9.09)	19 (5.34)	103 (10.45)	**0.004**
Three concave sign	39 (2.91)	9 (2.53)	30 (3.04)	0.620
Asymmetric lung sounds	251 (18.7)	98 (27.53)	153 (15.52)	**<0.001**
Rales				0.957
No rales	565 (42.1)	148 (41.57)	417 (42.29)	
Dry rales	87 (6.48)	24 (6.74)	63 (6.39)	
Moist rales	690 (51.42)	184 (51.69)	506 (51.32)	
Pleural effusion	54 (4.02)	23 (6.46)	31 (3.14)	**0.006**
Pulmonary consolidation	213 (15.87)	89 (25)	124 (12.58)	**<0.001**
Extrapulmonary complications				
Gastrointestinal dysfunction	115 (8.57)	42 (11.8)	73 (7.4)	**0.011**
Rash	21 (1.56)	7 (1.97)	14 (1.42)	0.476
Laboratory findings				
WBC, ×10^9/L	8.1 ± 3.6	8.1 ± 3.7	8.2 ± 3.6	0.605
NEUT, ×10^9/L	5.0 ± 2.9	5.2 ± 3.1	4.9 ± 2.9	0.161
LYMPH, ×10^9/L	2.4 ± 1.5	2.1 ± 1.3	2.5 ± 1.5	**<0.001**
EO, ×10^9/L	0.2 ± 0.3	0.2 ± 0.2	0.2 ± 0.3	0.242
RBC, ×10^12/L	4.6 ± 3.5	4.6 ± 0.4	4.7 ± 4.1	0.601
HGB, g/L	123 ± 10.3	123 ± 10.0	122 ± 10.4	0.223
RDW-CV, %	12.9 ± 2.1	12.8 ± 0.9	12.9 ± 2.4	0.339
RDW-SD, fL	38.2 ± 8.7	37.9 ± 2.3	38.4 ± 10.1	0.142
PLT, ×10^9/L	290 ± 99.0	276 ± 91.0	295 ± 101	**0.002**
hs-CRP, mg/L	19.1 ± 20.9	22.2 ± 25.0	17.9 ± 19.1	**0.003**
PCT, ng/ml	0.2 ± 0.9	0.2 ± 0.7	0.2 ± 0.9	0.871
IL-6, pg/ml	24.5 ± 116	39.2 ± 226	19.4 ± 22.8	0.121
LDH, U/L	313 ± 88.0	315 ± 105	312 ± 81.1	0.557
AST, U/L	37.5 ± 16.6	37.6 ± 26.3	37.4 ± 11.2	0.938
ALT, U/L	20.1 ± 15.7	21.1 ± 23.3	19.8 ± 11.8	0.327
ESR, mm/h	39.9 ± 19.6	37.4 ± 18.7	40.7 ± 19.9	**0.013**
Clinical outcomes				
Length of hospital stay (days)	6.0 ± 1.9	6.4 ± 2.3	5.8 ± 1.8	**<0.001**
SMPP	345 (25.71)	102 (28.65)	243 (24.65)	0.138
Mixed infection	450 (33.53)	117 (32.87)	333 (33.77)	0.756
Therapy				
Bronchoscopy therapy	12 (0.89)	4 (1.12)	8 (0.81)	0.592
Oxygen therapy	111 (8.27)	43 (12.08)	68 (6.9)	**0.002**
Methylprednisolone	443 (33.01)	154 (43.26)	289 (29.31)	<0.001

Data are shown as means (SD) or n (%). Significant differences in characteristics between the two groups are highlighted in bold. ALT, alanine transaminase; AST, aspartate transaminase; EO, erythrocyte; ESR, erythrocyte sedimentation rate; HGB, hemoglobin; hs-CRP, high-sensitivity C-reactive protein; IL-6, interleukin-6; LDH, lactate dehydrogenase; LYMPH, lymphocyte; MUMPP, macrolide-unresponsive *Mycoplasma pneumoniae* pneumonia; NEUT, neutrophil; PCT, procalcitonin; PLT, platelet; RBC, red blood cell; RDW-CV, red blood cell distribution width - coefficient of variation; RDW-SD, red blood cell distribution width - standard deviation; SMPP, severe Mycoplasma pneumoniae pneumonia; WBC, white blood cell.

We further performed multivariable logistic regressions to assess the risk factors of MUMPP, and found that age, ESR, length of hospital stay, and bad cough were associated with MUMPP after adjustment for sex, days of fever, wheezing, asymmetric lung sounds, pleural effusion, pulmonary consolidation, gastrointestinal dysfunction, severe pneumonia, NEUT, LYMPH, RDW-SD, PLT, hs-CRP, IL-6, and oxygen therapy (**Supplementary Table 1**). Older age was correlated with a higher risk of MUMPP (OR (95% CI) = 1.147 (1.074, 1.225), *P* < 0.001). In addition, longer duration of hospital stay and bad cough were also associated with higher risk of MUMPP (OR (95% CI) = 1.117 (1.031, 1.210), *P* = 0.007, and OR (95% CI) = 1.681 (1.228, 2.302), *P* = 0.001, respectively). However, higher level of ESR was correlated with a lower risk of MUMPP (OR (95% CI) = 0.986 (0.977, 0.994), *P* < 0.001).

### Laboratory-clinical discordance between mutated MPP and MUMPP

Out of 140 throat swab samples, 121 were found to carry macrolide resistance mutations in the 23S rRNA gene, and all were A2063G transitions. The proportion of mutated MPP was 86.43% (121/140) (**Table 2**).

**Table 2:** The laboratory-clinical discordance between mutated MPP and MUMPP.

	Mutated MPP		Total
MUMPP	No	Yes	
No	13 (9.29)	37 (26.43)	50 (35.71)
Yes	6 (4.29)	84 (60.00)	90 (64.29)
Total	19 (13.57)	121 (86.43)	140

MPP, *Mycoplasma pneumoniae* pneumonia; MUMPP, macrolide-unresponsive *Mycoplasma pneumoniae* pneumonia.

However, among the 140 patients, 50 patients received prescriptions of azithromycin and/or cephalosporin and/or penicillin, whereas the other 90 patients underwent treatment with doxycycline or moxifloxacin. The proportion of MUMPP was 64.29% (90/140) (**Table 2**).

There was significant laboratory-clinical discordance between mutated MPP and MUMPP (*P* < 0.0001).

### Comparison of patients with SMPP and non-SMPP

Among the 1342 cases of *M. pneumoniae* infection, 345 (25.71%) were diagnosed with SMPP. The comparison of clinical characteristics and laboratory findings with non-SMPP and SMPP were presented in **Supplementary Table 2**. No significant differences in sex was observed between the non-SMPP and SMPP groups (*P* = 0.090). Patients with SMPP were younger than those with non-SMPP (*P* < 0.001). Approximately 40% patients with SMPP were <3 years old, while more than 40% patients with non-SMPP were ≥7 years old. Patients with SMPP tended to have shorter duration of fever, longer duration of cough and hospital stay, and were more likely to have bad cough and dry rales and moist rales compared to those with non-SMPP (*P* < 0.05). No significant differences in the rates of MUMPP and mixed infection were observed between the non-SMPP and SMPP groups (*P*>0.05). As for laboratory findings, patients with SMPP had significantly higher WBC, LYMPH, PLT, LDH and ALT than those with non-SMPP. What’s more, patients with SMPP were more likely to treated with Moxifloxacin and Methylprednisolone (*P* = 0.005 and *P* < 0.001, respectively).

The results of multivariable logistic regressions showed that risk factors of SMPP included age, days of fever, days of cough, length of hospital stay, bad cough, asymmetric lung sounds, and dry rales after adjustment for sex, fever (>39°C), WBC, EO, HGB, PLT, LDH, AST, ALT, and antibacterial therapy (**Table 3**). Age and days of fever were negatively associated with the risk of developing SMPP, while the other factors were positively associated with SMPP. Notably, among the numerous risk factors, bad cough and dry rales have the highest odds ratios, which are 2.380 ((95% CI) = (1.812, 3.125), *P* < 0.001) and 4.969 ((95% CI) = (2.944, 8.387), *P* < 0.001), respectively.

**Table 3:** Multivariable logistic regressions results of association between various factors and SMPP.

	OR (95% CI)	*P* value
Sex (ref: boys)	0.849 (0.646, 1.115)	0.239
Age	0.884 (0.834, 0.937)	**<0.001**
Days of fever	0.923 (0.872, 0.977)	**0.006**
Fever (>39°C)	1.279 (0.925, 1.769)	0.137
Days of cough	1.021 (1.003, 1.039)	**0.019**
Bad cough	2.380 (1.812, 3.125)	**<0.001**
Asymmetric lung sounds	1.484 (1.048, 2.101)	**0.026**
Rales		
No rales	Ref	
Dry rales	4.969 (2.944, 8.387)	**<0.001**
Moist rales	1.253 (0.931, 1.685)	0.137
WBC	0.988 (0.946, 1.032)	0.581
EO	1.092 (0.669, 1.780)	0.726
HGB	0.999 (0.985, 1.013)	0.840
PLT	1.002 (1.000, 1.003)	0.062
LDH	1.001 (0.999, 1.003)	0.249
AST	0.995 (0.983, 1.007)	0.382
ALT	1.005 (0.996, 1.015)	0.287
Length of hospital stay (days)	1.177 (1.093, 1.267)	**<0.001**
Antibacterial therapy		
Azithromycin	Ref	
Azithromycin+Cephalosporin/Penicillin	0.966 (0.690, 1.352)	0.838
Doxycycline	0.884 (0.573, 1.363)	0.578
Moxifloxacin	1.438 (0.898, 2.304)	0.131

Significant risk factors of SMPP are highlighted in bold. ALT, alanine transaminase; AST, aspartate transaminase; EO, erythrocyte; HGB, hemoglobin; LDH, lactate dehydrogenase; PLT, platelet; SMPP, severe *Mycoplasma pneumoniae* pneumonia; WBC, white blood cell.

### Comparison of patients with pure MPP and those accompanied by multiple co-pathogens

A total of 450 patients with MPP were accompanied by multiple co-pathogens. The distribution of the types of co-pathogens is shown in **Supplementary Table 3**. Among the various respiratory pathogens, the five most common viruses are human rhinovirus, respiratory syncytial virus, infuenza virus A, parainfuenza virus, and adenovirus, and the three most common bacteria are *Haemophilus influenzae*, *Moraxella catarrhalis*, *Streptococcus pneumoniae*.

The clinical characteristics of patients with pure MPP and those accompanied by multiple co-pathogens were compared (**Supplementary Table 4**). MPP patients accompanied by multiple co-pathogens were younger than those pure MPP patients (*P* < 0.001). Again, no significant differences in sex was observed between the two groups. Patients accompanied by multiple co-pathogens were more likely to have shorter duration of fever (*P* = 0.004), but longer duration of cough (*P* < 0.001). Unexpectedly, patients accompanied by multiple co-pathogens were less likely to have fever (*P* = 0.027). The other symptoms and extrapulmonary manifestations were not significantly different between the two groups. Laboratory findings of patients with pure MPP and those accompanied by multiple co-pathogens were also compared. MPP patients accompanied by multiple co-pathogens tended to have higher levels of WBC, NEUT, LYMPH, PLT, and lower levels of EO than those with pure MPP (all *P* < 0.01). No significant differences in the rates of SMPP and MUMPP were observed between the two groups (*P*>0.05).

The results of multivariable logistic regressions showed that age, EO, and three concave sign were negatively associated with the risk of mixed infection, while days of cough, WBC, and PCT were positively associated with the risk of mixed infection, with adjustment for sex, days of fever, fever (>39°C), rales, RDW-CV, and PLT (**Table 4**).

**Table 4:** Multivariable logistic regressions results of association between various factors and MPP accompanied by multiple co-pathogens.

	OR (95% CI)	*P* value
Sex (ref: boys)	0.886 (0.696, 1.128)	0.325
Age	0.937 (0.889, 0.988)	**0.016**
Days of fever	0.960 (0.914, 1.008)	0.100
Fever (>39°C)	0.816 (0.615, 1.082)	0.158
Days of cough	1.028 (1.009, 1.046)	**0.003**
Three concave sign	0.317 (0.137, 0.733)	**0.007**
Rales		
No rales		
Dry rales	1.429 (0.862, 2.368)	0.167
Moist rales	1.066 (0.823, 1.380)	0.630
WBC	1.086 (1.039, 1.136)	**<0.001**
LYMPH	0.984 (0.875, 1.107)	0.791
EO	0.212 (0.097, 0.463)	**<0.001**
RDW-CV	1.009 (0.957, 1.064)	0.748
PLT	1.000 (0.999, 1.002)	0.810
PCT	1.383 (1.038, 1.844)	**0.027**

Significant risk factors of MPP accompanied by multiple co-pathogens are highlighted in bold. EO, erythrocyte; LYMPH, lymphocyte; MPP, *Mycoplasma pneumoniae* pneumonia; PCT, procalcitonin; PLT, platelet; RDW-CV, red blood cell distribution width - coefficient of variation; WBC, white blood cell.

## Discussion

The present study offers a comprehensive exploration of MPP after the COVID-19 pandemic based on two-year clinical data (2023-2024) from a tertiary hospital in Shenzhen, China. The findings provide valuable insights into the evolution of the epidemiological, clinical, and macrolide-resistant patterns of MPP, as well as the associated risk factors of MUMPP, SMPP, and co-pathogen infections, which are crucial for optimizing clinical treatment strategies.

### Epidemiological patterns and contributing factors of the recent *M. pneumoniae* epidemic

Our study revealed a distinct bimodal distribution of MPP cases during the 2023–2024 period. The incidence of MPP began to rise significantly in April 2023, peaked in November of the same year, and then gradually declined until March 2024. Following this, it rose again with the arrival of warmer weather, reaching a second smaller peak in August 2024. The first peak may reflect the post-NPI rebound, while the second peak may correspond to a persistent transmission in the context of seasonality and school cycles, as suggested by the generally lower incidence reported throughout 2024 compared to 2023.

Pneumonia caused by *M. pneumoniae* typically exhibits a distinct seasonality. However, the COVID-19 pandemic and the corresponding control measures have significantly disrupted this pattern. Previous studies commonly documented that *M. pneumoniae* infections predominantly occurred in the autumn and winter in northern China and peaked in the summer and autumn in southern China before the COVID-19 pandemic (Wang et al., 2022). However, recent studies from Guangzhou (Li et al., 2024), Shanghai (Zhu et al., 2024), Beijing (Gong et al., 2024), and Chongqing (You et al., 2024) reported that after the NPI discontinuation in December 2022, the incidence of MPP in mainland China generally increased significantly in the summer of 2023, peaked from September to December 2023, and then decreased markedly. And the incidence throughout 2024 was lower than that in 2023. Moreover, this trend was consistent with the epidemiological trends observed in North American and European countries in the northern hemisphere, where an increase in cases also began in the summer of 2023 and peaked in the winter of the same year (Edens et al., 2024; Edouard et al., 2024).

We propose that the recent *M. pneumoniae* epidemic from 2023 to 2024 may be a continuation of the 2019 epidemic. The global *M. pneumoniae* epidemic in late 2019 and early 2020 came to an abrupt halt due to the implementation of strict COVID-19 control measures. Before the COVID-19 pandemic, macrolide-resistant *M. pneumoniae* (MRMP) showed high resistance in East Asia, especially in China, Japan, and South Korea, compared to lower resistance in other regions, such as Europe and the United States, and the most common mutation in clinical MRMP patients is at position 2063 (A2063G) (Yang et al., 2025). After the COVID-19 pandemic, it remained high macrolide resistance rate in East Asia, and the most common mutation remained the A2063G mutation. For example, Xing et al. (2024) reported that 85% of *M. pneumoniae*-positive specimens had mutations detected at the 23S rRNA gene, with 99.7% showing A2063G mutation. This result was highly consistent with our findings: the proportion of mutated MPP was 86.43% (121/140), and all mutated MPP cases carried the A2063G mutation. Another study in Guangzhou, China, also found that among 236 clinical *M. pneumoniae* patients, 215 harbored macrolide resistance mutations in the 23 S rRNA gene, and all were A2063G transitions, with a MRMP proportion was 91.1% (215/236) in 2023 (Li et al., 2024). Additionally, in South Korea, Lee et al. (2024) report that the macrolide resistance rate remained high at 87.0%. Again, all identified mutations associated with macrolide resistance were characterized by the A2063G substitution (Lee et al., 2024). In contrast, in the United States and Europe, the macrolide resistance rates of *M. pneumoniae* have been relatively low after the COVID-19 pandemic. In 2023, the macrolide resistance rate in the United States was 7.14% (Edens et al., 2024), and in Denmark, it was 3.6% (Nordholm et al., 2024). In southeast Germany, the macrolide resistance rate of *M. pneumoniae* strains from 2023 to 2024 was 2.6% (Dumke, 2024). However, the most common mutation is still the change of A to G at location 2063 of the gene (Leber et al., 2025). The fact that the proportion of macrolide resistance and the major macrolide resistance genes remained unchanged before and after the COVID-19 pandemic, and the sudden interruption of the 2019 *M. pneumoniae* epidemic provides supporting evidence for our hypothesis (Meyer Sauteur et al., 2022; Meyer Sauteur and Beeton, 2024b).

Additionally, the phenomenon of “immune debt” accumulated during the three years of the COVID-19 pandemic may have played a crucial role in the *M. pneumoniae* epidemic (Yang et al., 2023; Lee et al., 2024). During this period, children were exposed to fewer pathogens due to social distancing and other control measures, resulting in a decline in their immune responses. This thus increased their susceptibility to common respiratory pathogens, including *M. pneumoniae*.

The unique biological characteristics of *M. pneumoniae*, such as its slow generation time (6 hours), long incubation period (1–3 weeks), and low transmission rate, also contributed to the epidemic pattern. These characteristics mean that *M. pneumoniae* requires a longer time to re-establish infection in the population. After the removal of COVID-19 restrictions, these factors may have facilitated the resurgence and spread of *M. pneumoniae*.

### Macrolide resistance laboratory-clinical discordance in MPP and possible reasons and mechanisms

In our study, 140 cases were included in tNGS testing, and the laboratory detection revealed a high macrolide resistance gene positive rate of 86.43% (121/140). However, among the 140 cases, the clinical use of doxycycline or moxifloxacin accounted for only 64.29% (90/140). Moreover, among all 1342 cases, only 26.53% (356/1342) needed to be treated with doxycycline or moxifloxacin. Similarly, a study in Chongqing found that among 155 children with MPP carrying the A2063/2064G mutation, only 28.39% (44/155) received quinolone therapy, with no tetracycline used (Cheng et al., 2024). Another study from Shanghai reported an 80% positive rate for macrolide resistance genes A2063G and A2064G among 434 tested patients, yet only 31.3% (155/494) of MPP patients were treated with quinolones or tetracyclines (Zhang et al., 2024). Internationally, the situation is comparable. In South Korea, the macrolide resistance gene detection positive rate was 87.0% for MPP children, with only 55.7% (54/97) eventually treated with second-line drugs (Lee et al., 2024). Therefore, despite the high positive rate of macrolide resistance genes, the actual proportion of patients treated with second-line drugs (including quinolones, doxycyclines, etc.) in clinical practice is much lower than the resistance gene positive rate (He et al., 2024). These studies indicate a discrepancy between the macrolide resistance gene positive rate and the actual clinical utilization rate of second-line antibiotics. Macrolide antibiotics still exhibit a certain level of effectiveness in clinical applications.

Except the macrolide resistance gene detection, there were also *in vitro* culture of *M. pneumoniae* and antimicrobial susceptibility testing using minimum inhibitory concentrations (MICs) to evaluate the antibiotic sensitivity of *M. pneumoniae* isolates. A study from Capital Institute of Pediatrics, Beijing, China reported that the resistance rates of *M. pneumoniae* isolates against erythromycin and azithromycin were both 100% (62/62), with A2063G mutation presented in all isolates (100%) (Jia et al., 2024). However, only seven patients did not get better after the treatment of macrolide antibiotics, and switched to the usage of levofloxacin, doxycycline, or cephalosporin (Jia et al., 2024). This also indicates that the results of *in vitro* antimicrobial susceptibility testing are inconsistent with the clinical efficacy.

Both macrolide resistance gene detection and *in vitro* antimicrobial susceptibility testing results demonstrate discordance with clinical treatment efficacy. We propose two main explanations for this observation: Firstly, the detection of macrolide resistance mutations (domain V of the 23S rRNA) in *M. pneumoniae* does not always correlate with clinical treatment failure. Secondly, clinicians may refrain from prescribing doxycycline or moxifloxacin due to specific clinical considerations—even when resistance is confirmed: (1) Age Restrictions: Doxycycline is generally avoided in children under the age of 8 due to its effects on developing teeth, and quinolone drugs (e.g., moxifloxacin) are explicitly prohibited for use in children under the age of 18. (2) Self-limiting nature of infection: A significant proportion of *M. pneumoniae* infections are mild and self-limiting, warranting only symptomatic care or no antimicrobial therapy. (3) Alternative clinical considerations: Treatment choices are influenced by factors such as drug availability, cost, patient allergies, side-effect profiles, severity of illness, and local guidelines, which are not solely determined by the presence of a resistance gene.

In the present study, we tend to consider that there is a discrepancy between the detection of resistance-related gene mutations in *M. pneumoniae* and the actual clinical efficacy of macrolide antibiotics. Firstly, macrolide antibiotics can achieve high concentrations in lung tissue and cells (Di Paolo et al., 2002). Even in the presence of resistance gene mutations, the local drug concentration in the lungs may still be sufficient to inhibit *M. pneumoniae*. For example, azithromycin can reach concentrations in lung tissue that are several times higher than those in the blood (Di Paolo et al., 2002). Moreover, the metabolism of azithromycin in children is slow and the clearance rate is low. After one or two courses of treatment, the concentration of azithromycin can be maintained at a relatively high level to inhibit *M. pneumoniae*. This high concentration may compensate for the reduced drug affinity caused by resistance mutations, thereby maintaining a certain level of antibacterial activity.

Secondly, macrolide antibiotics possess immune modulatory effects in addition to their antibacterial properties (Parnham, 2005; Friedlander and Albert, 2010; National Health Commission of the People’s Republic of China. Guidelines for the diagnosis and treatment of Mycoplasma pneumoniae pneumonia in children (2023 edition), 2023). They can regulate the host’s immune response and mitigate excessive inflammation. In *M. pneumoniae* infections, excessive immune reactions play a crucial role in the pathogenesis. Therefore, even in patients with MRMP, macrolide antibiotics may still alleviate symptoms and improve clinical outcomes through their immune modulatory effects. Besides, the results of antimicrobial susceptibility testing suggest that the clinical efficacy of azithromycin *in vivo* and *in vitro* are different, which may also be related to the various immune levels of different person.

Thirdly, the macrolide resistance mutations in *M. pneumoniae* may be heterogeneous. Not all strains exhibit complete resistance, and some may only harbor partial mutations, leading to relatively low resistance levels (National Health Commission of the People’s Republic of China. Guidelines for the diagnosis and treatment of Mycoplasma pneumoniae pneumonia in children (2023 edition), 2023). Additionally, mixed infections with both resistant and susceptible strains may occur. In such cases, macrolide antibiotics can still exert effects on the susceptible strains, resulting in certain therapeutic outcomes.

At the molecular level, the macrolide resistance in *M. pneumoniae* is primarily attributed to mutations in domain V of the 23S rRNA gene, with the A2063G mutation being the most common (National Health Commission of the People’s Republic of China. Guidelines for the diagnosis and treatment of Mycoplasma pneumoniae pneumonia in children (2023 edition), 2023). However, even with the A2063G mutation, macrolide antibiotics may still bind non-specifically to other regions of the ribosome, or affect other physiological processes of *M. pneumoniae*. These processes include other stages of protein synthesis or cell membrane function, thereby exerting inhibitory effects. Moreover, the ribosome of *M. pneumoniae* may exist in multiple conformations, and resistance mutations may only affect the drug binding in certain conformations, while the drug can still bind effectively in other conformations.

### Strengths and limitations

The present study covers a two-year period from 2023 to 2024, providing a relatively long-term and comprehensive perspective on the epidemiological patterns and clinical characteristics of MPP in the post-pandemic era. Additionally, the study strictly follows the Chinese Guidelines for the diagnosis and treatment of MPP in children (2023 edition) to diagnose MPP, MUMPP and SMPP, ensuring the accuracy and reliability of the diagnostic criteria. Furthermore, the study conducts a thorough analysis of various factors associated with MUMPP, SMPP, and co-pathogen infections, including clinical characteristics, laboratory findings, and treatment outcomes. This multifaceted analysis helps deepen the understanding of MPP and offers evidence-based guidance for clinical practice.

Despite the strengths of our study, there are some limitations. First, as a retrospective study, there might be potential biases in data collection and selection of patients. Second, the study was conducted in a single center, which might limit the generalizability of the findings to other populations. Third, only a subset of patients underwent tNGS for detecting macrolide resistance mutations, which might introduce selection bias toward patients with more severe disease, prolonged illness duration, or higher socioeconomic status. Therefore, our results derived from this subgroup should be interpreted with more caution, avoiding overgeneralization to the whole MPP population. Four, the detection of co-pathogens was based on nucleic acid tests, which cannot distinguish between active infections and colonization. Five, although we adjusted for multiple confounding factors, other potential confounders such as prior antibiotic exposure and socio-economic factors still existed. Future studies with prospective cohort design, larger sample sizes, and multi-center collaborations are needed to further validate our findings and explore the complex relationships between macrolide resistance detection and clinical outcome discordance in MPP.

## Conclusion

In conclusion, our study provides novel insights into MPP following the relaxation of NPIs. We found that MPP cases showed a bimodal temporal distribution from 2023 to 2024. Notably, there was a significant discordance between mutated MPP and MUMPP, as not all MPP patients with macrolide resistance mutations presented with clinical treatment failure. In other words, macrolides may still be effective in some cases. We also identified several risk factors for MUMPP, including older age, longer hospital stay and bad cough. Additionally, approximately 25% of MPP cases develop into SMPP, and co-pathogen infections were common in MPP patients. Distinct clinical and laboratory features were observed between MUMPP and non-MUMPP, SMPP and non-SMPP, co-pathogen infections and pure MPP. This study highlights the complex epidemiological and clinical landscape of MPP after the COVID-19 pandemic, and provides evidence for optimizing clinical treatment strategies.

## Data Availability

The raw data supporting the conclusions of this article will be made available by the authors, without undue reservation.
