# Supplementary material for: Clinical characteristics of hospitalized children with mycoplasma pneumoniae pneumonia in Chinese tertiary hospitals during the 2023–2024 post-pandemic period
Source: Front Cell Infect Microbiol. 2026 Mar 24;16:1652640. doi: 10.3389/fcimb.2026.1652640 (PMC13055549; doi:10.3389/fcimb.2026.1652640)
Supplement: Supplementary file 1 [file Table1.docx]

**Supplementary Tables**

**Table S1. Multivariate logistic regressions results of association between various factors and MUMPP**

|  | **OR (95% CI)** | ***P* value** |
| --- | --- | --- |
| Sex (ref: boys) | 1.192 (0.877, 1.62) | 0.262 |
| Age | 1.147 (1.074, 1.225) | **<0.001** |
| Days of fever | 1.004 (0.94, 1.071) | 0.916 |
| Fever (>39℃) | 1.069 (0.729, 1.567) | 0.732 |
| Hyperpyrexia (>40℃) | 0.906 (0.613, 1.34) | 0.621 |
| Bad cough | 1.681 (1.228, 2.302) | **0.001** |
| Wheezing | 0.609 (0.287, 1.294) | 0.198 |
| Asymmetric lung sounds | 1.185 (0.784, 1.792) | 0.421 |
| Pleural effusion | 1.392 (0.623, 3.111) | 0.42 |
| Pulmonary consolidation | 1.499 (0.973, 2.309) | 0.066 |
| Gastrointestinal dysfunction | 1.348 (0.694, 2.616) | 0.378 |
| Severe pneumonia | 0.893 (0.464, 1.716) | 0.733 |
| NEUT | 1.041 (0.982, 1.103) | 0.18 |
| LYMPH | 0.973 (0.825, 1.149) | 0.75 |
| RDW-SD | 0.95 (0.884, 1.022) | 0.167 |
| PLT | 1 (0.998, 1.002) | 0.775 |
| hs-CRP | 1.001 (0.993, 1.009) | 0.832 |
| IL-6 | 1.005 (0.999, 1.011) | 0.126 |
| ESR | 0.986 (0.977, 0.994) | **0.002** |
| Length of hospital stay (days) | 1.117 (1.031, 1.21) | **0.007** |
| Oxygen therapy | 1.567 (0.779, 3.152) | 0.208 |

Significant risk factors of MUMPP are highlighted in bold. Abbreviations: ESR, erythrocyte sedimentation rate; hs-CRP, high-sensitivity C-reactive protein; IL-6, interleukin-6; LYMPH, lymphocyte; MUMPP, macrolide-unresponsive *Mycoplasma pneumoniae* pneumonia; NEUT, neutrophil; PLT, platelet; RDW-SD, red blood cell distribution width - standard deviation.

**Table S2. Clinical and laboratory characteristics of patients with SMPP and non-SMPP**

|  | **SMPP**  **(N=345)** | **non-SMPP**  **(N=997)** | ***P* value** |
| --- | --- | --- | --- |
| Sex (male) | 193 (55.94) | 505 (50.65) | 0.090 |
| Age (months) | 5.3±3.1 | 6.5±2.8 | <0.001 |
| <=1 | 21 (6.09) | 20 (2.01) |  |
| 1~3 | 115 (33.33) | 172 (17.25) |  |
| 4~6 | 103 (29.86) | 368 (36.91) |  |
| >=7 | 106 (30.72) | 437 (43.83) |  |
| **Symptom** |  |  |  |
| Days of fever | 4.5±2.7 | 5.1±2.5 | <0.001 |
| Fever (>39℃) | 225 (65.22) | 693 (69.51) | 0.140 |
| Hyperpyrexia (>40℃) | 82 (23.84) | 206 (20.75) | 0.229 |
| Days of cough | 8.5±8.8 | 6.9±6.6 | 0.003 |
| Bad Cough | 183 (53.04) | 316 (31.7) | <0.001 |
| Asymmetric lung sounds | 76 (22.03) | 175 (17.55) | 0.066 |
| Rales |  |  | <0.001 |
| No rales | 106 (30.72) | 459 (46.04) |  |
| Dry rales | 54 (15.65) | 33 (3.31) |  |
| Moist rales | 185 (53.62) | 505 (50.65) |  |
| **Laboratory findings** |  |  |  |
| WBC, ×10^9/L | 8.7±4.0 | 7.9±3.4 | 0.002 |
| NEUT, ×10^9/L | 5.2±3.4 | 4.9±2.7 | 0.109 |
| LYMPH, ×10^9/L | 2.7±1.7 | 2.3±1.4 | <0.001 |
| EO, ×10^9/L | 0.2±0.5 | 0.2±0.2 | 0.064 |
| RBC, ×10^12/L | 4.6±0.4 | 4.7±4.0 | 0.419 |
| HGB, g/L | 122±9.4 | 123±10.5 | 0.198 |
| RDW-CV, % | 12.9±1.2 | 12.8±2.3 | 0.323 |
| RDW-SD, fL | 38.8±16.7 | 38.0±2.2 | 0.380 |
| PLT, ×10^9/L | 309±110 | 283±93.9 | <0.001 |
| hs-CRP, mg/L | 19.1±21.8 | 19.1±20.6 | 0.967 |
| PCT, ng/ml | 0.3±1.4 | 0.2±0.6 | 0.283 |
| IL-6, pg/ml | 24.0±36.7 | 24.6±133 | 0.903 |
| LDH, U/L | 327±97.4 | 308±84.0 | <0.001 |
| AST, U/L | 39.0±19.8 | 36.9±15.3 | 0.084 |
| ALT, U/L | 21.9±19.0 | 19.5±14.3 | **0.032** |
| ESR, mm/h | 39.4±19.0 | 40.0±19.9 | 0.638 |
| **Clinical outcome** |  |  |  |
| Length of hospital stay (days) | 6.5±2.2 | 5.8±1.8 | <0.001 |
| MUMPP | 102 (29.57) | 254 (25.48) | 0.138 |
| Mixed infection | 116 (33.62) | 334 (33.5) | 0.967 |
| **Therapy** |  |  |  |
| Antibacterial therapy |  |  | **0.005** |
| Azithromycin | 105 (30.43) | 343 (34.4) |  |
| Azithromycin+Cephalosporin/Penicillin | 138 (40) | 400 (40.12) |  |
| Doxycycline | 49 (14.2) | 167 (16.75) |  |
| Moxifloxacin | 53 (15.36) | 87 (8.73) |  |
| Methylprednisolone | 154 (44.64) | 289 (28.99) | <0.001 |

Data are shown as means (SD) or n (%). Significant differences in characteristics between the two groups are highlighted in bold. Abbreviations: ALT, alanine transaminase; AST, aspartate transaminase; EO, erythrocyte; ESR, erythrocyte sedimentation rate; HGB, hemoglobin; hs-CRP, high-sensitivity C-reactive protein; IL-6, interleukin-6; LDH, lactate dehydrogenase; LYMPH, lymphocyte; NEUT, neutrophil; PCT, procalcitonin; PLT, platelet; RBC, red blood cell; RDW-CV, red blood cell distribution width - coefficient of variation; RDW-SD, red blood cell distribution width - standard deviation; SMPP, severe *Mycoplasma pneumoniae* pneumonia; WBC, white blood cell.

**Table S3. Co-pathogen infection of patients with *M. pneumoniae***

|  | **n** | **%** |
| --- | --- | --- |
| **Virus** |  |  |
| Human rhinovirus | 96 | 7.15% |
| Respiratory syncytial virus | 56 | 4.17% |
| Infuenza virus A | 38 | 2.83% |
| Parainfuenza virus | 38 | 2.83% |
| Adenovirus | 32 | 2.38% |
| Human metapneumovirus | 8 | 0.60% |
| Enterovirus | 8 | 0.60% |
| SARS-CoV-2 | 7 | 0.52% |
| Infuenza virus B | 5 | 0.37% |
| Human bocavirus | 5 | 0.37% |
| **Bacteria** |  |  |
| *Haemophilus influenzae* | 142 | 10.58% |
| *Moraxella catarrhalis* | 64 | 4.77% |
| *Streptococcus pneumoniae* | 44 | 3.28% |
| *Bordetella pertussis* | 20 | 1.49% |
| *Streptococcus intermedius* | 2 | 0.15% |
| **Chlamydia** | 1 | 0.07% |

**Table S4. Clinical characteristics of patients with pure MPP and those accompanied by multiple co-pathogens**

|  | **MPP accompanied by multiple co-pathogens**  **(N=450)** | **Pure MPP**  **(N=892)** | ***P* value** |
| --- | --- | --- | --- |
| Sex (male) | 243 (54) | 455 (51.01) | 0.301 |
| Age (months) | 5.7±2.8 | 6.5±2.9 | **<0.001** |
| <=1 | 14 (3.11) | 27 (3.03) |  |
| 1~3 | 124 (27.56) | 163 (18.27) |  |
| 4~6 | 169 (37.56) | 302 (33.86) |  |
| >=7 | 143 (31.78) | 400 (44.84) |  |
| **Symptoms** |  |  |  |
| Days of fever | 4.6±2.9 | 5.1±2.4 | **0.004** |
| Fever (>39℃) | 290 (64.44) | 628 (70.4) | **0.027** |
| Hyperpyrexia (>40℃) | 97 (21.65) | 191 (21.48) | 0.944 |
| Days of cough | 8.5±8.9 | 6.7±6.2 | **<0.001** |
| Bad Cough | 158 (35.11) | 341 (38.23) | 0.265 |
| Wheezing | 45 (10) | 77 (8.63) | 0.411 |
| Three concave sign | 8 (1.78) | 31 (3.48) | 0.081 |
| Asymmetric lung sounds | 76 (16.89) | 175 (19.62) | 0.226 |
| Rales |  |  | 0.109 |
| No rales | 175 (38.89) | 390 (43.72) |  |
| Dry rales | 36 (8) | 51 (5.72) |  |
| Moist rales | 239 (53.11) | 451 (50.56) |  |
| Pleural effusion | 12 (2.67) | 42 (4.71) | 0.072 |
| Pulmonary consolidation | 68 (15.11) | 145 (16.26) | 0.588 |
| **Extrapulmonary manifestations** |  |  |  |
| Gastrointestinal dysfunction | 33 (7.33) | 82 (9.19) | 0.251 |
| Rash | 8 (1.78) | 13 (1.46) | 0.655 |
| **Laboratory findings** |  |  |  |
| WBC, ×10^9/L | 9.1±4.3 | 7.7±3.1 | **<0.001** |
| NEUT, ×10^9/L | 5.6±3.5 | 4.7±2.5 | **<0.001** |
| LYMPH, ×10^9/L | 2.6±1.5 | 2.3±1.4 | **<0.001** |
| EO, ×10^9/L | 0.1±0.2 | 0.2±0.3 | **0.004** |
| RBC, ×10^12/L | 4.9±6.0 | 4.5±0.4 | 0.256 |
| HGB, g/L | 122±10.0 | 123±10.4 | 0.601 |
| RDW-CV, % | 12.9±1.0 | 12.8±2.5 | 0.183 |
| RDW-SD, fL | 38.1±2.4 | 38.3±10.5 | 0.691 |
| PLT, ×10^9/L | 302±108 | 284±93.7 | **0.002** |
| hs-CRP, mg/L | 19.3±22.1 | 18.9±20.2 | 0.775 |
| PCT, ng/ml | 0.4±1.4 | 0.2±0.3 | **0.005** |
| IL-6, pg/ml | 32.3±198 | 20.5±21.9 | 0.228 |
| LDH, U/L | 313±84.8 | 312±89.6 | 0.864 |
| AST, U/L | 37.3±11.9 | 37.6±18.5 | 0.736 |
| ALT, U/L | 19.7±10.4 | 20.4±17.8 | 0.388 |
| ESR, mm/h | 39.8±19.7 | 39.9±19.6 | 0.950 |
| **Clinical outcomes** |  |  |  |
| Length of hospital stay (days) | 6.1±2.0 | 5.9±1.9 | 0.238 |
| SMPP | 116 (25.78) | 229 (25.67) | 0.967 |
| MUMPP | 117 (26) | 239 (26.79) | 0.756 |
| **Therapy** |  |  |  |
| Bronchoscopy therapy | 5 (1.11) | 7 (0.78) | 0.549 |
| Oxygen therapy | 41 (9.11) | 70 (7.85) | 0.428 |
| Antibacterial therapy |  |  | **0.001** |
| Azithromycin | 124 (27.56) | 324 (36.32) |  |
| Azithromycin+Cephalosporin/Penicillin | 209 (46.44) | 329 (36.88) |  |
| Doxycycline | 65 (14.44) | 151 (16.93) |  |
| Moxifloxacin | 52 (11.56) | 88 (9.87) |  |
| Methylprednisolone | 138 (30.67) | 305 (34.19) | 0.195 |

Data are shown as means (SD) or n (%). Significant differences in characteristics between the two groups are highlighted in bold. Abbreviations: ALT, alanine transaminase; AST, aspartate transaminase; EO, erythrocyte; ESR, erythrocyte sedimentation rate; HGB, hemoglobin; hs-CRP, high-sensitivity C-reactive protein; IL-6, interleukin-6; LDH, lactate dehydrogenase; LYMPH, lymphocyte; MPP, *Mycoplasma pneumoniae* pneumonia; MUMPP, macrolide-unresponsive *Mycoplasma pneumoniae* pneumonia; NEUT, neutrophil; PCT, procalcitonin; PLT, platelet; RBC, red blood cell; RDW-CV, red blood cell distribution width - coefficient of variation; RDW-SD, red blood cell distribution width - standard deviation; SMPP, severe Mycoplasma pneumoniae pneumonia; WBC, white blood cell.
